# Identification of a highly active tannase enzyme from the oral pathogen *Fusobacterium nucleatum* subsp. *polymorphum*

**DOI:** 10.1186/s12934-018-0880-4

**Published:** 2018-02-26

**Authors:** Julen Tomás-Cortázar, Laura Plaza-Vinuesa, Blanca de las Rivas, José Luis Lavín, Diego Barriales, Leticia Abecia, José Miguel Mancheño, Ana M. Aransay, Rosario Muñoz, Juan Anguita, Héctor Rodríguez

**Affiliations:** 1 Macrophage and Tick Vaccine Laboratory, CIC bioGUNE, Derio, Bizkaia Spain; 2 Genome Analysis Platform, CIC bioGUNE, Derio, Bizkaia Spain; 3 Laboratorio de Biotecnología Bacteriana, Instituto de Ciencia y Tecnología de los Alimentos y Nutrición (ICTAN)-Consejo Superior de Investigaciones Científicas (CSIC), Madrid, Spain; 4 Departamento de Cristalografía y Biología Estructural, Instituto de Química-Física “Rocasolano” (IQFR-CSIC), Madrid, Spain; 5 Centro de Investigación Biomédica en Red de enfermedades hepáticas y digestivas (CIBERehd), Instituto de Salud Carlos III, Madrid, Spain; 6 Ikerbasque, Basque Foundation for Science, Bilbao, Bizkaia Spain

**Keywords:** Phenolics biotransformation, Tannase, Industrial, Fusobacterium, Oral pathogen

## Abstract

**Background:**

Tannases are tannin-degrading enzymes that have been described in fungi and bacteria as an adaptative mechanism to overcome the stress conditions associated with the presence of these phenolic compounds.

**Results:**

We have identified and expressed in *E. coli* a tannase from the oral microbiota member *Fusobacterium nucleatum* subs. *polymorphum* (TanB_Fnp_). TanB_Fnp_ is the first tannase identified in an oral pathogen. Sequence analyses revealed that it is closely related to other bacterial tannases. The enzyme exhibits biochemical properties that make it an interesting target for industrial use. TanB_Fnp_ has one of the highest specific activities of all bacterial tannases described to date and shows optimal biochemical properties such as a high thermal stability: the enzyme keeps 100% of its activity after prolonged incubations at different temperatures up to 45 °C. TanB_Fnp_ also shows a wide temperature range of activity, maintaining above 80% of its maximum activity between 22 and 55 °C. The use of a panel of 27 esters of phenolic acids demonstrated activity of TanB_Fnp_ only against esters of gallic and protocatechuic acid, including tannic acid, gallocatechin gallate and epigallocatechin gallate. Overall, TanB_Fnp_ possesses biochemical properties that make the enzyme potentially useful in biotechnological applications.

**Conclusions:**

We have identified and characterized a metabolic enzyme from the oral pathogen *Fusobacterium nucleatum* subsp. *polymorphum*. The biochemical properties of TanB_Fnp_ suggest that it has a major role in the breakdown of complex food tannins during oral processing. Our results also provide some clues regarding its possible participation on bacterial survival in the oral cavity. Furthermore, the characteristics of this enzyme make it of potential interest for industrial use.

**Electronic supplementary material:**

The online version of this article (10.1186/s12934-018-0880-4) contains supplementary material, which is available to authorized users.

## Background

Tannins are high molecular weight secondary phenolic metabolites of plant origin that have been traditionally considered antinutrients due to their capacity to bind and precipitate protein [1, 2]. This group of chemicals can be found in tea, wine, berries, fruits and chocolate among other dietary components. They are toxic compounds for a variety of microorganisms because of their protein and iron binding capacity, and may interfere with many biological processes that are essential for their growth [1]. In turn, some microorganisms have developed strategies to overcome tannin toxicity, including the presence of genes in their genomes encoding degrading enzymes such as tannase enzymes [3]. Initially, tannases, also known as tannin acyl hydrolases (EC 3.1.1.20), were described in fungi and studied primarily because of their industrial use in processes related to food detannification and the removal of pollutants, in the leather industry or for the production of gallic acid, which is an important intermediate in the synthesis of the antibiotic trimethoprim [3, 4]. Recently, tannases have also been isolated from bacteria that populate environments rich in vegetable content, although just a few of the genes encoding bacterial tannases have been described and even fewer have been characterized biochemically. The most studied tannases are present in bacteria isolated from the rumen, gut microbiota or soils with abundant vegetation [5–11]. Two different types of tannases have been described so far that are encoded by two different genes. Extracellular tannases (encoded by the *tanA* gene) contain a signal peptide and a molecular size of around 66 kDa. These include TanA_Sl_ from *Staphylococcus lugdunensis* [5, 9, 12, 13], TanA_Lp_ from *Lactobacillus plantarum* [8], and TanA_Sg_ from *Streptococcus gallolyticus* [14]. In addition, 50 kDa intracellular bacterial tannases (encoded by *tanB* genes) have also been described. These proteins are found in *L. plantarum* (TanB_Lp_) [6, 15] or *S. gallolyticus* (TanB_Sg_) [7]. Recently, an extracellular tannase that is nevertheless encoded by a *tanB* gene has been described in *Streptomyces sviceus* (TanB_Ss_) [11].

The gut and the oral cavity harbor distinctive populations of bacteria permanently exposed to a vast array of chemical compounds present in food, including tannins. The human microbiota is a massive and largely unexplored source of enzymes with new and/or improved activities [16]; however, limited research has been performed to identify microbes capable of degrading tannins within the inhabitants of the human body. Although bacteria that contain enzymes with tannase activity, such as *L. plantarum*, *S. gallolyticus*, or *S. lugdunensis*, have been described in the human gastrointestinal tract [9, 17–19], many questions remain about the oral metabolism of food tannins. As the oral cavity is continuously exposed to tannins, we hypothesized that oral bacteria might harbor tannin-degrading genes in their genomes. Previously, a tannase (TanA_Ap_) from *Atopobium parvulum*, a species abundant in the oral cavity, was characterized [20]. This protein exhibited the lowest specific activity among bacterial tannases and was unable to hydrolyze complex tannins. Therefore, the biochemical properties of TanA_Ap_ discarded its role in the breakdown of complex food tannins. Herein, we have identified and characterized the first tannase enzyme described within the genus *Fusobacterium* and overall, from a pathogenic bacterium. The study of its biochemical properties and the substrates among different relevant tannin derivatives present in food demonstrate that it is among the most active tannases described so far possessing a wide range of substrate specificity that includes several derivatives of gallic acid, protocatechuic acid and complex tannins.

## Results

### Presence of a putative tannase in members of the genus *Fusobacterium*

**Fig. 1:**
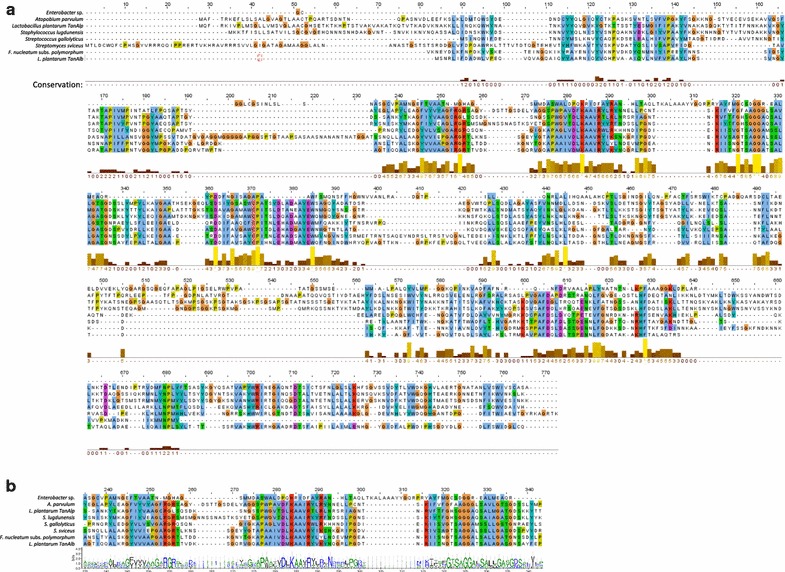
Comparison of TanB_Fnp_ with previously described bacterial tannases. **a** Alignment of the whole protein sequences of bacterial tannases showing conserved regions. **b** Alignment of conserved motifs in bacterial tannases and sequence logos depicting the consensus sequence and diversity of bacterial tannase sequences. The sequences corresponding to those domains predicted to have hydrolase activity and with the highest conservation scores were used to identify a consensus sequence. The color scheme is defined by hydrophobicity scale (amino acids representation default), where each color corresponds to the following code: hydrophilic residues (RKDENQ) in blue, neutral residues (SGHTAP) in green, and hydrophobic (YVMCLFIW) in black

**Fig. 2:**
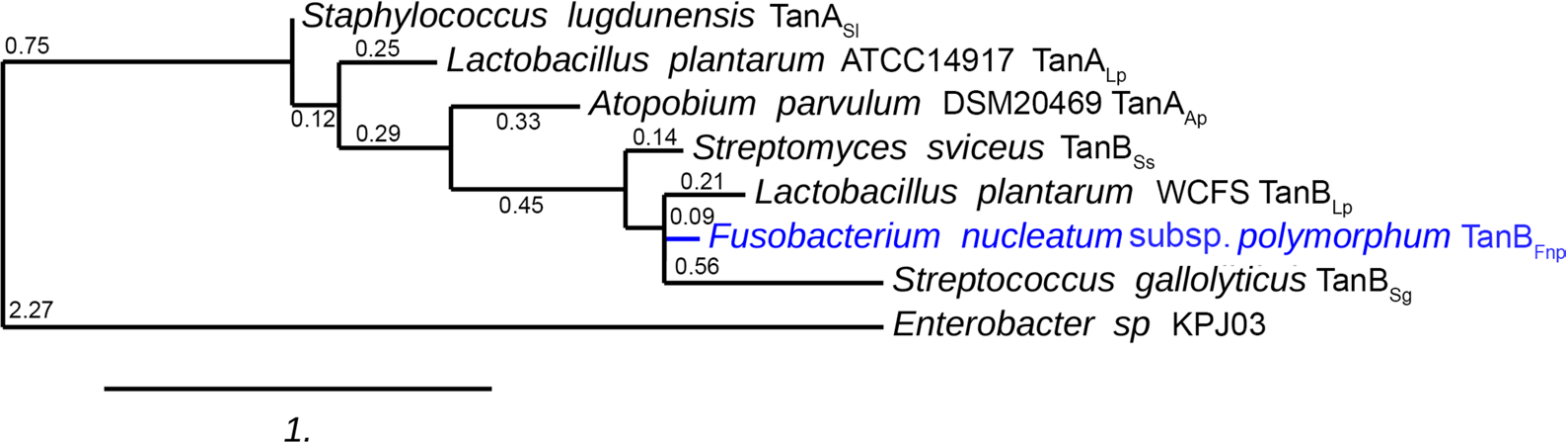
Phylogenetic tree of bacterial tannases. Tree showing evolutionary relationships between all bacterial tannases genetically identified to date. The numbers indicate branches lengths. *F. nucleatum* subsp. *polymorphum* TanB_Fnp_ is indicated in blue

A search for *L. plantarum* tannase (TanB_Lp_) homologues was performed in common oral bacteria genera using NCBI blastx. Among the hits, a gene from *Fusobacterium nucleatum* subsp. *polymorphum* (annotated as a hypothetical protein) showed 44% identity to TanB_Lp_ (GMHT-1603-MONOMER from BioCyc database) [21]. Because of the identity, we annotated the gene as tanB_Fnp_ and the protein that it encodes as TanB_Fnp_. In addition, the CDD web tool revealed that TanB_Fnp_ presented domains conserved among bacterial tannases including the essential amino acids for hydrolytic-esterase activity previously described as the active site of the protein [22]. Alignment of the whole amino acid sequence and a partial alignment showing just key residues are showed in Fig. 1. The presence of these key residues identified using Python’s WebLogo package (Fig. 1b) suggested that the *F. nucleatum* subsp. *polymorphum* gene product is a relevant candidate to have tannase activity. We also performed a phylogenetic analysis of this gene with other tannases previously identified in order to get further information about their proximity. The dendrogram in Fig. 2 shows that TanB_Fnp_ is more similar to *L. plantarum* TanB_Lp_ tannase than to all the bacterial tannases previously described. We also analyzed the distribution of orthologs of the *F. polymorphum* tannase in species that belong to the *Fusobacterium* genus and other oral bacteria. Orthologs for TanB_Fnp_ were found in all *F. nucleatum* subspecies with the exception of the subspecies *fusiforme* as well as in other *Fusobacterium* species related to oral and gut diseases, such as *F. periodonticum*, *F. necrophorum*, *F. massiliense* and *F. hwasooki.* Strikingly, more than 30% of the total number of TanB_Fnp_ orthologs detected were found among oral bacteria species, including the most prevalent genera identified in the human oral microbiota (*Prevotella*, *Neisseria*, *Streptococcus*, *Fusobacterium* and *Haemophilus*) [23] (Additional file 1: Table S1).

### TanB_Fnp_ is among the most active bacterial tannases identified to date

**Fig. 3:**
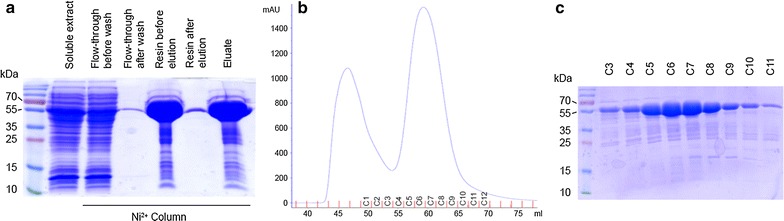
Purification of recombinant *F. nucleatum subsp. polymorphum* tannase TanB_Fnp_. **a** SDS-PAGE analysis of TanB_Fnp_ fractions obtained before and after His-affinity resin chromatography. **b** UV absorbance of different fractions obtained after gel filtration chromatography. **c** SDS-PAGE analysis of fractions obtained after gel filtration chromatography

**Fig. 4:**
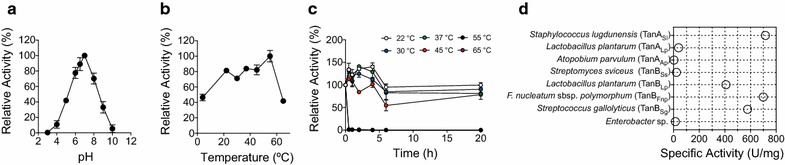
Biochemical properties of TanB_Fnp_. **a** Relative enzymatic activity of TanB_Fnp_ using methyl gallate as substrate over a range of pH. The observed maximum activity was defined as 100%. **b** Relative enzymatic activity of TanB_Fnp_ over a range of temperatures. The observed maximum activity was defined as 100%. **c** Relative enzymatic activity of TanB_Fnp_ after preincubation of the purified protein at 22, 30, 37, 45, 55 and 65 °C for the indicated lengths of time. **d** Absolute enzymatic activity of TanB_Fnp_ compared to previously described tannases using methyl gallate as substrate. The experiments were performed in triplicate. The values shown represent the mean value and standard error

The *Fusobacterium* putative tannase TanB_Fnp_ is a 58 kDa protein with an alkaline isoelectric point (theoretical pI, 8.9) and a predicted signal peptide comprising the 20 initial residues. The presence of a signal peptide is not a common characteristic among TanB type tannases. In order to avoid solubility issues, the gene was cloned without the signal peptide into the pHis-parallels II expression vector and purified using His-affinity chromatography combined with a gel filtration step (Fig. 3). In order to address whether TanB_Fnp_ was, in fact, a tannase enzyme, a colorimetric assay was performed. Rhodanine assay [24] indirectly detects tannase activity over methyl gallate by measuring colorimetrically at 520 nm the binding of the vicinal hydroxilic groups of the reaction product, gallic acid, with rhodanine (see “Methods”). Rhodanine reacts specifically with gallic acid while it does not bind other phenol compounds. Once the activity was confirmed, the absolute activity of TanB_Fnp_ was measured, using *Lactobacillus plantarum* TanB_Lp_ as a reference. The activity of TanB_Fnp_ using methyl gallate as a substrate was one of the highest described to date in bacteria (699 U/mg) being equivalent to a recently isolated *Streptococcus lugdunensis* enzyme (716 U/mg), and 76.5% higher than TanB_Lp_, the reference used in the same experiment (395.9 U/mg). TanB_Fnp_ activity was more than 20% higher than the one described for *Streptococcus gallolyticus*, TanB_Sg_ [7] (Fig. 4d).

### Biochemical characterization of TanB_Fnp_

We then determined the biochemical properties of TanB_Fnp_ using the purified protein. Its optimum activity pH was measured at 30 °C in 50 mM phosphate buffer. Figure 4a shows an optimum pH for TanB_Fnp_ of 7 and suboptimal activity of the enzyme at pH values ranging from 6 to 8, although it still retained around 80% of the maximal activity. The optimal activity temperature was determined incubating the protein in 50 mM phosphate buffer pH 6.5. TanB_Fnp_ showed the highest activity at 55 °C, albeit it retained around 80% of the maximal activity at all temperatures tested between 22 and 55 °C (Fig. 4b). The thermal stability of the enzyme was also determined by pre-incubation at different temperatures ranging from 22 to 65 °C for increasing lengths of time up to 20 h, followed by activity determination. Surprisingly, pre-incubation of the enzyme between 22 and 45 °C did not substantially affect the activity of the enzyme, maintaining methyl gallate hydrolysis rates above 60% of the maximum activity and in some cases, increasing the enzymatic activity. On the contrary, incubation at temperatures higher than 45 °C induced a dramatic decrease in TanB_Fnp_ activity after 30 min of incubation (Fig. 4c).

We also tested the influence of distinct additives on TanB_Fnp_ activity (Table 1). The addition of HgCl_2_ and β-mercaptoethanol abolished the activity of the enzyme whereas the rest of the compounds tested increased the hydrolization of the substrate to different extents. This effect was especially significant for Triton X-100 (242%), EDTA (206%) and KCl (191%).

**Table 1:** Relative activity of TanB_Fnp_ in the presence of different additives

Additive (1 mM)	Relative activity (%)
None (control)	100
EDTA	206
KCl	191
HgCl_2_	0
CaCl_2_	126
MgCl_2_	177
ZnCl_2_	126
Triton X-100	242
DMSO	158
Tween 80	169
Urea	104
β-mercaptoethanol	1.7

### Substrate specificity of TanB_Fnp_

**Fig. 5:**
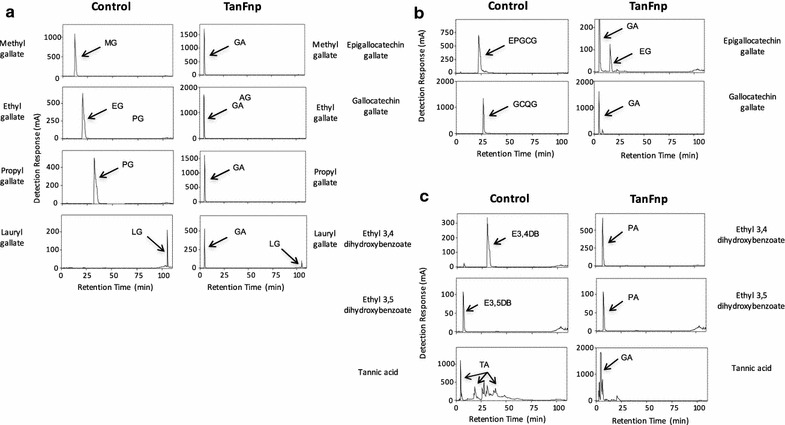
TanB_Fnp_ substrate specifity. Activity of TanB_Fnp_ on gallic acid esters (**a**), protocatechuic acid esters (**b**) and complex tannins (**c**). The substrates used were: *MG* methyl gallate, *EG* ethyl gallate, *PG* propyl gallate, *LG* lauryl gallate, *EPGCG* epigallocatechin gallate, *GCQG* gallocatechin gallate, *E3,4DB* ethyl 3,4-dihydroxybenzoate, *E3,5DB* ethyl 3,5-dihydroxybenzoate, *TA* tannic acid. The products generated were: *GA* gallic acid, *PA* protocatechuic acid

In order to identify relevant food substrates for TanB_Fnp_, the purified enzyme was incubated with different esters of phenolic acids and the reaction products were then analyzed using high-performance liquid chromatography coupled with a diode array detection unit (HPLC–DAD). As a control, all the reactions were also performed and analyzed in the absence of the enzyme (see “Methods”). TanB_Fnp_ hydrolyzed several simple esters of gallic acid (3,4,5-trihydroxybenzoic acid) regardless of the length of their aliphatic alcohol. Among this group of compounds, methyl gallate, ethyl gallate, propyl gallate and lauryl gallate (Fig. 5a) were transformed into gallic acid by TanB_Fnp_. Other substrates, including polyphenolic esters of gallic acid such as gallocatechin gallate and epigallocatechin gallate were also hydrolyzed (Fig. 5b). The ability of the enzyme to break complex natural tannins was also studied using tannic acid as a substrate. This complex phenolic compound was almost entirely hydrolyzed by TanB_Fnp_, generating a simpler mix of compounds with gallic acid as the predominant product (Fig. 5c). Finally, esters of protocatechuic acid such as ethyl 3,4-dihydroxybenzoate and ethyl 3,5-dihydroxybenzoate were also degraded by the enzyme, being both converted into protocatechuic acid (Fig. 5c). These results demonstrated that TanB_Fnp_ is active against a wide spectrum of gallic and protocatechuic acid-derived substrates. Table 2 shows the battery of compounds tested in this study and whether they were hydrolyzed by the enzyme.

**Table 2:** Activity of TanB_Fnp_ against different substrates

Substrate	Activity	Products detected
Methyl gallate	YES	Gallic acid
Ethyl gallate	YES	Gallic acid
Propyl gallate	YES	Gallic acid
Lauryl gallate	YES	Gallic acid
Methyl benzoate	NO	–
Ethyl benzoate	NO	–
Methyl 4-hydroxybenzoate	NO	–
Ethyl 4-hydroxybenzoate	NO	–
Methyl 2,4-dihydroxybenzoate	NO	–
Methyl 2,5-dihydroxybenzoate	NO	–
Ethyl 3,4-dihydroxybenzoate	YES	Protocatechuic acid
Ethyl 3,5-dihydroxybenzoate	YES	Protocatechuic acid
Methyl vanillate	NO	–
Chlorogenic acid	NO	–
Methyl salicitate	NO	–
Methyl ferulate	NO	–
Ethyl ferulate	NO	–
Ellagic acid	NO	–
Methyl caffeate	NO	–
Methyl sinapinate	NO	–
Methyl *p*-coumarate	NO	–
Tannin	YES	Gallic acid
Catechin	NO	–
Galocatechin	NO	–
Epigallocatechin	NO	–
Epigallocatechin gallate	YES	Gallic acid
Gallocatechin gallate	YES	Gallic acid

## Discussion

The search for new tannase enzymes with improved activity and new substrate specificities constitutes a permanent focus for industrial microbiology research due to their extensive use in the food and pharmaceutical industries. Indeed, the search for enzymes with tannase activity has recently been expanded to the human microbial population. Herein, we describe a new tannase enzyme from the oral pathogen *F. nucleatum* susbp. *polymorphum.* Oral bacteria need to have extensive metabolic resources to face the presence of food components with antimicrobial properties that they may encounter. We previously described a tannase from *A. parvulum*, an inhabitant of the human oral cavity [20]. However, this tannase (TanA_Ap_) possessed low specific activity and was unable to hydrolyze complex tannins. Therefore, it is unlikely that this tannase contributes to tannin breakdown. Because the presence of enzymes with tannase activity is one of the preferred mechanisms to overcome phenolic-related stress, we sought and identified a homologous protein of the tannase from *L. plantarum*, TanB_Lp_, within the *Fusobacterium* genus. Phylogenetically, TanB_Fnp_ shows a closer relationship with TanB_Lp_ than with all other bacterial tannases. TanB_Lp_ has been previously associated with a cluster of tannases unrelated to those of fungal origin [8]. Therefore, TanB_Fnp_ might be included within the same cluster of bacterial tannin-degrading enzymes.

The biochemical characterization of TanB_Fnp_ reveals that it is an enzyme with potential industrial applications. Its activity against methyl gallate is among the highest described in bacteria so far and considerably higher than other tannases previously reported [7, 8, 10, 11, 15, 20]. Other biotechnological features that are important for its potential industrial application include its temperature range of activity. The optimal temperature of most tannases varies between 30 and 40 °C [4]. Strikingly, TanB_Fnp_ is highly active in a much wider range of temperatures, from 22 to 60 °C, with a maximum activity peak at 60 °C, similar to *S. gallolyticus* (TanB_Sg_) and *S. sviceus* tannases (TanB_Ss_) [7, 11]. Accordingly, TanB_Fnp_ thermal stability is much higher than that of all previously described tannases. Similar to TanB_Sg_, TanB_Fnp_ is able to keep high activity rates after it has been exposed for long time intervals to temperatures ranging from 22 to 45 °C while most bacterial tannases suffer a deep decrease in their activity after long exposures to temperatures equal to or higher than 37 °C. As previously suggested [11], it is possible that for extracellular tannases, such as TanB_Fnp_, a better adaptability to the harsh extracellular environment has induced the development of more resistant enzymes. Both the high performance temperature and its thermal resistance to unfolding or denaturalization in the absence of substrate are key features that support TanB_Fnp_ biotechnological use. These characteristics may likely improve its interaction with substrates, favor high mass transfer rates, and lower the risk of contamination [25].

Like all bacterial tannases characterized to date, TanB_Fnp_ showed the highest activity rates at pH between 6 and 8, which overlaps the pH of the oral cavity (between 6.7 and 7.3). The neutral pH range of activity seems to be a common characteristic of bacterial tannases in contrast with those of fungal origin that present activity peaks under acidic conditions [26].

The search for enzymes with new activities is also an important target in tannase research due to the high variety of tannin substrates. We performed a substrate characterization using 27 different compounds from different tannin families. The results presented are identical to the substrate specificity previously described for TanB_Lp_, the most similar tannase to TanB_Fnp_. While other tannases such as TanB_Sg_ and TanA_Lp_ only degrade esters of phenolic acid with short chain aliphatic alcohols [7, 8], TanB_Fnp_ is capable of degrading esters of phenolic acids with long chain alcohols such as lauryl gallate. The hydrolyzing activity of TanB_Fnp_ against longer esters points to a markedly different substrate-binding site in this enzyme that would permit the access of these bulkier compounds.

*Fusobacterium nucleatum* subsp. *polymorphum* is an inhabitant of the human oral cavity frequently isolated from dental plaque biofilms [27]. As a pathogen, it has been repeatedly associated with peridontitis and extraoral infections including preterm births and colorectal cancer [28]. However, little is known about the metabolic arsenal that allows them to thrive in these environments. The extracellular tannase identified in *F. nucleatum* susps. *polymorphum*, TanB_Fnp_, might provide an ecological advantage to bacteria thriving in a niche permanently exposed to phenolic stress from food sources. The enzyme is still highly active (80% of its optimal activity) at body temperature and at pH usually found within the human body. Vegetable food residues are permanently deposited in the oral cavity as a consequence of food intake. An extracellular tannase could be an important survival factor for *F. nucleatum* with a dual role during bacterial colonization of teeth: the detoxification of toxic compounds and the provision of a source of sugar moieties resulting from the hydrolyzation of complex tannins. The wide distribution of tannase enzymes among oral inhabitants in comparison to microbial species in other niches suggests that its presence may constitute an adaptative advantage to compete in an environment that is permanently receiving tannins from diet sources. Therefore, TanB_Fnp_ may constitute a putative virulence factor for *F. nucleatum* and a potential target of therapeutic intervention for this pathogen.

The description of TanB_Fnp_ increases our knowledge about tannin breakdown in the human gastrointestinal tract. Oral and intestinal tannases could contribute to tannin digestion. At least five different species of oral or intestinal bacteria have been described to possess active tannases, including *A. parvulum*, *L. plantarum*, *S. gallolyticus*, *S. lugdunensis* and, in this work, *Fusobacterium nucleatum* subsp. *polymorphum*. Further testing would be required to define the metabolism of these phenolic compounds comprehensively. In particular, the activity of the complex communities of microorganisms present in all parts of the human digestive tract would need to be examined. Moreover, as tannase-producing bacteria have been identified in several human cancer microbiomes [29], the study of the association between dietary tannin intake and tumor recurrence or regression, may be critical in understanding the role of gut bacteria on the anti-cancer effects of dietary polyphenols.

## Conclusions

In this work, we describe an enzyme with tannase activity in the oral pathogen *Fusobacterium nucleatum* subsp. *polymorphum*. TanB_FNP_ is one of the most active tannases described to date. This fact combined with an extraordinary thermal stability and a wide range of temperature activity makes TanB_FNP_ a suitable candidate for industrial applications. Moreover, since *F. nucleatum* subsp. *polymorphum* is a pathogen associated with oral and extra-oral diseases our research increases the knowledge about a putative niche adaptation determinant that might be involved in virulence associated with the transformation of diet antimicrobial compounds.

## Methods

### Bacterial strains and growth conditions

*Escherichia coli* strains DH5α and BL21(DE3) were used as transformation and expression hosts, respectively, for the pHISp-fusotan vector. The bacteria were grown in LB medium containing ampicillin (100 µg/mL) at 37 °C with agitation.

### Gene cloning

Genomic DNA from *F. nucleatum* subsp*. polymorphum* Strain F0401 was obtained from BEI Resources Repository. Standard molecular biology procedures [30] were followed to clone the tannase gene avoiding the signal peptide of the protein. The gene was PCR-amplified using phusion hot start II polymerase (Thermo Fisher Scientific, Waltham, MA) with primers *Fusotannase*-*f* (5′-CGC CAT GGG CGT AAA AAA TGA GTA TGA TT-3′) and *Fusotannase*-*r* (5′-CGG TCG ACT TAT TTT TTT ACA ACA CCA TC-3′). The PCR product was purified using a PCR purification kit (Thermo Fisher Scientific). The 1.5 kb PCR product and the vector pHis parallel 2 (Addgene) were digested with *Nco*I and *Sal*I and then ligated for 16 h using T4 DNA ligase (Promega, Madison, WI). The ligation mixture was transformed in DH5α cells and positive colonies were verified by colony PCR and sequencing. Positive plasmids (pHISp-fusotan) were finally transformed into *Escherichia coli* BL21(DE3) cells for protein production.

### Protein production and purification

Sequence-confirmed *TanB*_*Fnp*_ clones in *E. coli* BL21(DE3) were grown in Luria–Bertani medium supplemented with ampicillin and induced with 1 mM IPTG for 16 h at 20 °C. The His-tag fusion protein was then purified by nickel affinity chromatography (GE Healthcare, Uppsala, Sweden) and eluted in 20 mM Tris, pH 7.5 with 150 mM NaCl and 250 mM Imidazole. For a second purification step using gel filtration chromatography, fractions containing TanB_Fnp_ identified by SDS-PAGE were pooled, concentrated and loaded onto a HiLoad 10/300 GL Superdex 75 column (GE Healthcare) pre-equilibrated in 20 mM Tris pH 7.5; 150 mM NaCl, using an AKTA chromatography system (GE Healthcare). Fractions with the protein of interest were pooled and the protein was concentrated and stored at − 80 °C until its use. Protein concentration was determined using the BCA protein assay kit (Thermo Fisher Scientific).

### Determination of tannase activity

The generation of gallic acid in hydrolysis reactions was determined in triplicate with the following assay: TanB_Fnp_ (10 μg) in 700 μL of 50 mM phosphate buffer, pH 6.5, was incubated with 40 μL of 25 mM methyl gallate (1 mM final concentration) for 5 min at 37 °C. After incubation, 150 μL of a methanolic rhodanine solution (0.667% rhodanine in 100% methanol) was added to the reaction mixture. After 5 min of incubation at 30 °C, 100 μL of 0.5 M KOH was added and the absorbance at 520 nm was measured on a spectrophotometer. A standard curve using gallic acid concentrations ranging from 0.125 to 1 mM was prepared. One unit of tannase activity was defined as the amount of enzyme required to release 1 μmol of gallic acid per minute under standard reaction conditions.

### Biochemical characterization

Activities of Tan_Fnp_ from *F. nucleatum* subsp. *polymorphum* F401 were measured at 4, 20, 30, 37, 45, 55, and 65 °C to determine the optimal temperature for enzymatic activity. The optimum pH value for tannase activity was determined by studying its pH dependence within the pH range between 3 and 10. The buffers used were: acetic acid-sodium acetate buffer for pH 3–5, citric acid-sodium citrate for pH 6, sodium phosphate for pH 7, Tris–HCl for pH 8, glycine–NaOH for pH 9, and sodium carbonate-bicarbonate for pH 10. A 100 mM concentration was used in all the buffers. The rhodanine assay was used for the optimal pH characterization of TanB_Fnp_ [8]. Since the rhodanine-gallic acid complex forms only under basic conditions, after the enzymatic degradation of methyl gallate, 100 μL of 0.5 M KOH was added to the reaction mixture to ensure that the same pH value (pH 11) was achieved in all samples assayed.

For temperature stability measurements, TanB_Fnp_ was incubated in 50 mM phosphate buffer, pH 6.5, at 22, 30, 37, 45, 55, and 65 °C for 15 min, 30 min, and 1, 2, 5, and 18 h. After incubation, the residual activity was measured as described above.

To test the effects of metals and ions on the activity of TanB_Fnp_, the enzymatic activity was measured in the presence of different additives at a final concentration of 1 mM. The additives analyzed were MgCl_2_, KCl, CaCl_2_, HgCl_2_, ZnCl_2_, Triton X-100, urea, Tween 80, EDTA, dimethyl sulfoxide (DMSO) and β-mercaptoethanol. All the determinations were done in triplicate.

### TanB_Fnp_ substrate specificity

The activity of TanB_Fnp_ against 27 potential substrates was analyzed. The substrates assayed were gallic esters (methyl gallate, ethyl gallate, propyl gallate, and lauryl gallate), benzoic esters (methyl benzoate and ethyl benzoate), hydroxybenzoic esters (methyl 4-hydroxybenzoate, ethyl 4-hydroxybenzoate, propyl 4-hydroxybenzoate and butyl 4-hydroxybenzoate), a vanillic ester (methyl vanillate), dyhydroxybenzoic esters (methyl 2,4-dihydroxybenzoate, ethyl 3,4-dihydroxybenzoate or protocatechuic acid ethyl ester and ethyl 3,5-dihydroxybenzoate), a gentisic ester (methyl gentisate), a salicylic ester (methyl salicylate) and ferulic esters (ferulic methyl ester and ferulic ethyl ester). Tannic acid and epigallocatechin gallate were also assayed as potential substrates. Recombinant tannase (10 μg/mL) was incubated at 37 °C in ammonium acetate 50 mM, pH 5 in the presence of the substrate (1 mM). As controls, acetate buffers containing the reagents but not the enzyme were incubated under the same conditions. After incubation, the samples were analyzed by high-performance liquid chromatography with diode array detection (HPLC–DAD). A Thermo chromatograph (Thermo Fisher Scientific) equipped with a P4000 SpectraSystem pump, an AS3000 autosampler, and a UV6000LP photodiode array detector was used. A gradient of solvent A (water-acetic acid, 98:2 [vol/vol]) and solvent B (water-acetonitrile-acetic acid, 78:20:2 [vol/vol/vol]) was applied to a reversed-phase Nova-pack C18 cartridge (25 cm by 4.0-mm inside diameter [i.d.]; 4.6-μm particle size) at room temperature as follows: 0–55 min, 80% B linear, 1.1 mL/min; 55–57 min, 90% B linear, 1.2 mL/min; 57–70 min, 90% B isocratic, 1.2 mL/min; 70–80 min, 95% B linear, 1.2 mL/min; 80–90 min, 100% linear, 1.2 mL/min; 100–120 min, washing, 1.0 mL/min; and re-equilibration of the column under the initial gradient conditions. Samples were injected onto the cartridge after being filtered through a 0.45-μm PVDF filter. Detection of the substrates and the degradation compounds was performed spectrophotometrically by scanning from 220 to 380 nm. The identification of degradation compounds was carried out by comparing the retention times and spectral data of each peak with those of standards from commercial suppliers.

### Bioinformatic analyses

The sequences of tanases analyzed in this work were inspected for conserved functional domains with the CDD web tool [31]. Then, the region harboring the functional domain related to the tanase activity was extracted. These sequences were the input for a Multiple Sequence Analysis using CLUSTAL omega [32], in order to identify conserved amino acid patterns among them, and use it as input for the phylogenetic analysis carried out by the Phylogeny.fr web-service [33] including the following steps: the CLUSTAL omega alignment was used as input, followed by the removal of poorly aligned/gapped regions using Gblocks v0.91b [34] with default settings for both tools. Phylogenetic trees were reconstructed using the maximum likelihood method with the PhyML program v3.0 [35] using the WAG substitution model and 100 bootstrap replicates for inner branch accuracy. The graphical representation and edition of the phylogenetic tree was performed with TreeDyn v198.3 [36]. Sequence logos were obtained using Python’s WebLogo package [37] using the CLUSTAL omega’s alignment file as input. The scale of the logo was measured in bits, which are units of measure with a precise thermodynamic relationship to energy. Display error bars indicate an approximate Bayesian 95% confidence interval.

In order to study the distribution of tannase ortholog genes among *Fusobacterium* spp. (in dental plaque and gut microflora) and other oral bacteria, we performed a BLASTp [38] search against the non-redundant database limited to bacteria taxa. Results were filtered by e-value (< 1e−20). Those with a query coverage > 50% and sequence identity > 20% were retained as putative orthologs (Additional file 1: Table S1).

## Additional file

**Additional file 1: Table S1.** TanB_Fnp_ orthologs in bacterial species, including *Fusobacterium* and the most prevalent genera identified in the human oral microbiota.

## References

[1] Chung KT, Wong TY, Wei CI, Huang YW, Lin Y (1998). Tannins and human health: a review. Crit Rev Food Sci Nutr.

[2] Smeriglio A, Barreca D, Bellocco E, Trombetta D (2017). Proanthocyanidins and hydrolysable tannins: occurrence, dietary intake and pharmacological effects. Br J Pharmacol.

[3] Aguilar CN, Rodriguez R, Gutierrez-Sanchez G, Augur C, Favela-Torres E, Prado-Barragan LA, Ramirez-Coronel A, Contreras-Esquivel JC (2007). Microbial tannases: advances and perspectives. Appl Microbiol Biotechnol.

[4] Chávez-González M, Rodríguez-Duránb LV, Balagurusamy N, Prado-Barragán A, Rodríguez R, Contreras JC, Aguilar CN (2012). Biotechnological advances and challenges of tannase: an overview. Food Bioprocess Technol.

[5] Chaitanyakumar A, Anbalagan M (2016). Expression, purification and immobilization of tannase from *Staphylococcus lugdunensis* MTCC 3614. AMB Express.

[6] Iwamoto K, Tsuruta H, Nishitaini Y, Osawa R (2008). Identification and cloning of a gene encoding tannase (tannin acylhydrolase) from *Lactobacillus plantarum* ATCC 14917(T). Syst Appl Microbiol.

[7] Jimenez N, Barcenilla JM, de Felipe FL, de las Rivas B, Munoz R (2014). Characterization of a bacterial tannase from *Streptococcus gallolyticus* UCN34 suitable for tannin biodegradation. Appl Microbiol Biotechnol.

[8] Jimenez N, Esteban-Torres M, Mancheno JM, de las Rivas B, Munoz R (2014). Tannin degradation by a novel tannase enzyme present in some *Lactobacillus plantarum* strains. Appl Environ Microbiol.

[9] Noguchi N, Ohashi T, Shiratori T, Narui K, Hagiwara T, Ko M, Watanabe K, Miyahara T, Taira S, Moriyasu F, Sasatsu M (2007). Association of tannase-producing *Staphylococcus lugdunen*sis with colon cancer and characterization of a novel tannase gene. J Gastroenterol.

[10] Sharma KP, John PJ (2011). Purification and characterization of tannase and tannase gene from *Enterobacter* sp. Process Biochem.

[11] Wu M, Wang Q, McKinstry WJ, Ren B (2015). Characterization of a tannin acyl hydrolase from *Streptomyces sviceus* with substrate preference for digalloyl ester bonds. Appl Microbiol Biotechnol.

[12] Noguchi N, Fukuzawa M, Wajima T, Yokose K, Suzuki M, Nakaminami H, Kawai T, Moriyasu F, Sasatsu M (2017). Specific clones of *Staphylococcus lugdunensis* may be associated with colon carcinoma. J Infect Public Health.

[13] Noguchi N, Goto K, Ro T, Narui K, Ko M, Nasu Y, Utsumi K, Takazawa K, Moriyasu F, Sasatsu M (2010). Using the tannase gene to rapidly and simply identify *Staphylococcus lugdunensis*. Diagn Microbiol Infect Dis.

[14] Jimenez N, Reveron I, Esteban-Torres M, de Felipe FL, de las Rivas B, Munoz R (2014). Genetic and biochemical approaches towards unravelling the degradation of gallotannins by *Streptococcus gallolyticus*. Microb Cell Fact.

[15] Curiel JA, Rodriguez H, Acebron I, Mancheno JM, de las Rivas B, Munoz R (2009). Production and physicochemical properties of recombinant *Lactobacillus plantarum* tannase. J Agric Food Chem.

[16] Koppel N, Rekdal VM, Balskus EP (2017). Chemical transformation of xenobiotics by the human gut microbiota. Science.

[17] Abdulamir AS, Hafidh RR, Abu Bakar F (2011). The association of *Streptococcus bovis*/*gallolyticus* with colorectal tumors: the nature and the underlying mechanisms of its etiological role. J Exp Clin Cancer Res.

[18] Abdulamir AS, Hafidh RR, Mahdi LK, Al-jeboori T, Abubaker F (2009). Investigation into the controversial association of Streptococcus gallolyticus with colorectal cancer and adenoma. BMC Cancer.

[19] Rusniok C, Couve E, Da Cunha V, El Gana R, Zidane N, Bouchier C, Poyart C, Leclercq R, Trieu-Cuot P, Glaser P (2010). Genome sequence of *Streptococcus gallolyticus*: insights into its adaptation to the bovine rumen and its ability to cause endocarditis. J Bacteriol.

[20] Jimenez N, Santamaría L, Esteban-Torres M, De Las Rivas B, Muñoz R (2014). Contribution of a tannase from *Atopobium parvulum* DSM 20469T in the oral processing of food tannins. Food Res Int.

[21] Sanchez-Mangas R, Garcia-Ferrrer A, de Juan A, Arroyo AM (2010). The probability of death in road traffic accidents. How important is a quick medical response?. Accid Anal Prev.

[22] Ren B, Wu M, Wang Q, Peng X, Wen H, McKinstry WJ, Chen Q (2013). Crystal structure of tannase from *Lactobacillus plantarum*. J Mol Biol.

[23] Zhao H, Chu M, Huang Z, Yang X, Ran S, Hu B, Zhang C, Liang J (2017). Variations in oral microbiota associated with oral cancer. Sci Rep.

[24] Inoue KH, Hagerman AE (1988). Determination of gallotannin with rhodanine. Anal Biochem.

[25] Turner P, Mamo G, Karlsson EN (2007). Potential and utilization of thermophiles and thermostable enzymes in biorefining. Microb Cell Fact.

[26] Lekha PK, Lonsane BK (1997). Production and application of tannin acyl hydrolase: state of the art. Adv Appl Microbiol.

[27] Karpathy SE, Qin X, Gioia J, Jiang H, Liu Y, Petrosino JF, Yerrapragada S, Fox GE, Haake SK, Weinstock GM, Highlander SK (2007). Genome sequence of *Fusobacterium nucleatum* subspecies polymorphum—a genetically tractable fusobacterium. PLoS ONE.

[28] Han YW, Wang X (2013). Mobile microbiome: oral bacteria in extra-oral infections and inflammation. J Dent Res.

[29] Lopez de Felipe F, de las Rivas B, Munoz R (2014). Bioactive compounds produced by gut microbial tannase: implications for colorectal cancer development. Front Microbiol.

[30] Maniatis T, Fritsch EF, Sambrook J (1989). Molecular cloning: a laboratory manual.

[31] Marchler-Bauer A, Derbyshire MK, Gonzales NR, Lu S, Chitsaz F, Geer LY, Geer RC, He J, Gwadz M, Hurwitz DI (2015). CDD: NCBI’s conserved domain database. Nucleic Acids Res.

[32] Sievers F, Wilm A, Dineen D, Gibson TJ, Karplus K, Li W, Lopez R, McWilliam H, Remmert M, Soding J (2011). Fast, scalable generation of high-quality protein multiple sequence alignments using Clustal Omega. Mol Syst Biol.

[33] Dereeper A, Guignon V, Blanc G, Audic S, Buffet S, Chevenet F, Dufayard JF, Guindon S, Lefort V, Lescot M (2008). Phylogeny.fr: robust phylogenetic analysis for the non-specialist. Nucleic Acids Res.

[34] Castresana J (2000). Selection of conserved blocks from multiple alignments for their use in phylogenetic analysis. Mol Biol Evol.

[35] Anisimova M, Gascuel O (2006). Approximate likelihood-ratio test for branches: a fast, accurate, and powerful alternative. Syst Biol.

[36] Chevenet F, Brun C, Banuls AL, Jacq B, Christen R (2006). TreeDyn: towards dynamic graphics and annotations for analyses of trees. BMC Bioinform.

[37] Crooks GE, Hon G, Chandonia JM, Brenner SE (2004). WebLogo: a sequence logo generator. Genome Res.

[38] Camacho C, Coulouris G, Avagyan V, Ma N, Papadopoulos J, Bealer K, Madden TL (2009). BLAST+: architecture and applications. BMC Bioinform.

